# Safe Administration of anti-PD-L1 Atezolizumab in a Patient with Metastatic Urothelial Cell Carcinoma and End-Stage Renal Disease on Dialysis

**DOI:** 10.1155/2019/3452762

**Published:** 2019-02-10

**Authors:** Alessandro Parisi, Alessio Cortellini, Katia Cannita, Melissa Bersanelli, Corrado Ficorella

**Affiliations:** ^1^Medical Oncology, St. Salvatore Hospital, L'Aquila, Italy; ^2^Department of Biotechnological and Applied Clinical Sciences, University of L'Aquila, Via Vetoio, 67100 L'Aquila, Italy; ^3^Medical Oncology, University Hospital of Parma, Parma, Italy

## Abstract

Anti-PD-L1 (programmed cell death-ligand 1) agents, such as atezolizumab, have been approved for the treatment of different advanced cancers. However, no study enrolled end-stage renal disease (ESRD) patients, and to our knowledge, there are no significant published data about safety and efficacy of PD-L1 inhibitors in this population. Here, we report the first case of a male patient with metastatic urothelial cell carcinoma and ESRD on dialysis, safely treated with atezolizumab.

## 1. Background

Immune checkpoint inhibitors (ICIs), such as anti-PD-L1 (programmed cell death-ligand 1) agents, are now routine clinical practice in the treatment algorithm of several malignancies. Patients with impaired renal function have been mostly excluded from registrative clinical trials of these agents. The cohort 1 of the phase II trial of the anti-PD-L1 atezolizumab, as first-line therapy in cisplatin-ineligible patients with locally advanced or metastatic urothelial carcinoma (IMvigor210), included patients with mild to moderate chronic renal failure (CRF). Eighty-three among 119 enrolled patients (69.7%) had a glomerular filtration *rate* < 60 and > 30 mL/min at the baseline [1]. However, no study enrolled end-stage renal disease (ESRD) patients, and to our knowledge, there are no consistent published data about safety and efficacy of PD-L1 inhibitors in this population.

Here, we report the case of a male patient with metastatic urothelial cell carcinoma and ESRD on dialysis, safely treated with atezolizumab.

## 2. Case Report

We report the case of a male patient, a smoker, with a history of chronic obstructive lung disease, hypothyroidism, glaucoma, and bilateral mild carotid stenosis. In January 2016, he underwent cystoprostatectomy with resection of seminal vesicles and locoregional lymphadenectomy. The histopathological examination showed bladder sarcomatoid carcinoma, biphasic, with a superficial high-grade urothelial component and a sarcomatoid component deeply up to the fat tissue around the bladder, with metastasis in 4 out of 37 resected lymph nodes. Pathological stage IIIB (pT3b pN2-TNM, VII edition). Due to postoperative complications, the patient reached adjuvant treatment with cisplatin and gemcitabine, between May and July 2016, after a 4-month delay from surgery. Subsequent clinical and instrumental follow-up resulted negative, until February 2017, when a positron emission tomography scan (PET scan) showed a pathological enhancement of retroperitoneal and iliac lymph nodes and of the left adrenal gland. The iliac lymph node recurrence caused a rapid worsening of the renal function (creatinine reached 2.5 mg/dL in about a week), so the patient underwent the placement of bilateral percutaneous nephrostomy, with benefit.

**Figure 1:**
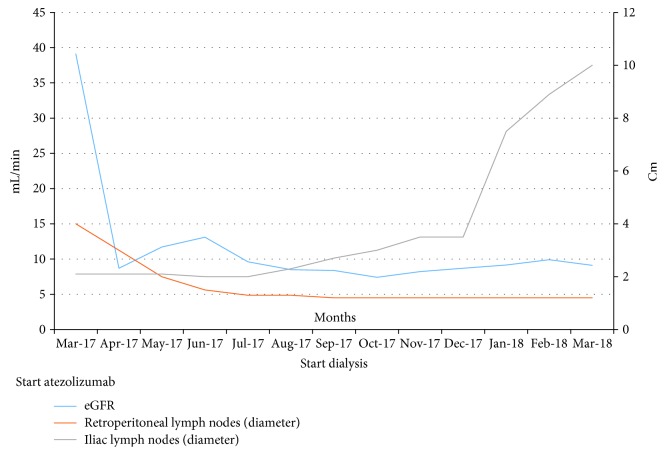
Timeline with the estimated glomerular filtration rate (eGFR) and diameters of target lymph nodal metastases.

In March 2017, given the discrete clinical conditions, with Eastern Cooperative Oncology Group performance status (ECOG-PS) 2 and mild renal impairment (creatinine: 1.5 mg/dL—ULN: 1.2 mg/dL), we enrolled the patient in the Italian expanded access program of atezolizumab. In April 2017, before the second administration of atezolizumab (standard dose of 1200 mg intravenous, every three weeks), the patient accidentally removed the left nephrostomy, developing a severe acute kidney injury, which hesitated in ESRD, despite replacement of bilateral nephrostomy and supportive therapy. The antinucleus antigen test was negative, and the urinary dipstick protein was 100 mg/dL (2+). The timing of renal impairment onset, immediately after nephrostomy removal, allowed us to assume with reasonable certainty that the renal injury was not immune-related, albeit without a biopsy confirmation, which the patient rejected. Despite the development of ESRD, we decided to continue treatment with atezolizumab, given the absence of therapeutic options and in agreement with the patient and his relatives.  Figure 1 summarized the timeline with the eGFR and diameters of target lymph node metastases.

**Figure 2:**
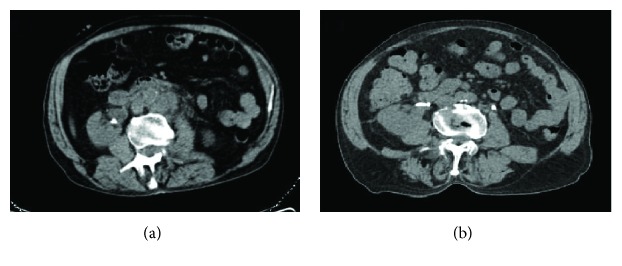
Computed tomography (CT) images (a) before and (b) after 3 administrations of atezolizumab showing a significant decrease in the size of lymph nodal metastasis.

In July 2017, the computed tomography (CT) scan without iodate contrast showed a partial response, with a volumetric reduction of about 30-70% of the lymph nodal and left adrenal gland metastases (Figure 2). The patient then continued treatment with atezolizumab with progressive clinical benefit and without significant toxicities, developing only grade 1 itching, asthenia, nausea, dysgeusia, and constipation (NCI CTCAE v. 4.0). He began dialysis treatment only at September 2017, due to difficulties in positioning the fistula. In October 2017, the CT scan showed disease progression to the right ischiopubic branch and with substantial stability of lymph nodal and adrenal gland metastases. Given this, we decided to continue atezolizumab beyond progression adding an adjuvant therapy with denosumab (120 mg subcutaneous injection, every 4 weeks). In January 2018, the CT scan evidenced progressive disease at the lymph nodes, with worsening of pain. A palliative radiation therapy (8 gray) at these sites allowed us to achieve better pain control and to still continue the treatment with atezolizumab. In March 2018, due to a nonpathological femoral fracture caused by severe coxarthrosis, the patient discontinued atezolizumab and underwent a total hip replacement. The last administration of atezolizumab dates back to February 2018; he died in May 2018, as a result of a diastatic perforation of the caecum (not related to atezolizumab).

## 3. Discussion and Conclusion

First-line chemotherapy for patients with cisplatin-ineligible locally advanced or metastatic urothelial carcinoma is associated with short response duration, poor survival, and high toxicity. Atezolizumab demonstrated encouraging durable response rates, survival, and tolerability in the phase II trial, also in patients with mild to moderate CRF and ECOG-PS 2 [1], while in the phase III trial, IMvigor 211 did not show a statistically significant benefit in overall survival over single agent chemotherapy [2]. Only few cases of ESRD patients treated with anti-PD-1 (programmed cell death-1) agents were previously reported [3–5] and none with anti-PD-L1.

Renal irAEs were reported to be rare and among the irAEs that occur later from the treatment beginning; indeed, they are reported to occur at least three months after the ICI commencement [6, 7]. So, the favorable safety profile of atezolizumab and the timing of the renal injury onset (soon after the treatment beginning) got us thinking that it was not drug-related and to continue the immunotherapy. Moreover, the patient would not have been a candidate for other chemotherapy regimens.

We consider the 12-month overall survival, the good tolerability, and the absence of pharmacological interactions with denosumab good clinical outcomes. Nevertheless, we are far from asserting that each cancer patient with ESRD on dialysis can be safely treated with ICIs, which have an intrinsic risk to lead to renal irAEs. The risk-benefit ratio should be carefully evaluated in clinical practice, according to the expected results and the patient priorities.

No registrative study with anti-PD-1/PD-L1 agents enrolled cancer patients with ESRD on dialysis, so there are no consistent data about safety and efficacy of these treatments in this population. This is the first case report of an advanced cancer patient (urothelial) with ESRD on dialysis, safely treated with atezolizumab. This report would be helpful for clinicians who had to administer anti-PD-L1 agents to cancer patients with ESRD.
